# Image reconstruction of oxidized cerebral cytochrome C oxidase changes from broadband near-infrared spectroscopy data

**DOI:** 10.1117/1.NPh.4.2.021105

**Published:** 2017-05-24

**Authors:** Sabrina Brigadoi, Phong Phan, David Highton, Samuel Powell, Robert J. Cooper, Jeremy Hebden, Martin Smith, Ilias Tachtsidis, Clare E. Elwell, Adam P. Gibson

**Affiliations:** a University College London, Department of Medical Physics and Biomedical Engineering, Biomedical Optics Research Laboratory, London, United Kingdom; b University of Padova, Department of Developmental and Social Psychology, Padova, Italy; c National Hospital for Neurology and Neurosurgery, Neurocritical Care, London, United Kingdom; d University College London, Department of Computer Science, London, United Kingdom; e NIHR University College London Hospitals Biomedical Research Centre, London, United Kingdom

**Keywords:** functional near-infrared spectroscopy, cytochrome C oxidase, image reconstruction, broadband, visual stimulation

## Abstract

In diffuse optical tomography (DOT), overlapping and multidistance measurements are required to reconstruct depth-resolved images of oxy- (${\mathrm{HbO}}_{2}$) and deoxy- (HHb) hemoglobin concentration changes occurring in the brain. These can be considered an indirect measure of brain activity, under the assumption of intact neurovascular coupling. Broadband systems also allow changes in the redox state of cytochrome c oxidase (oxCCO) to be measured, which can be an important biomarker when neurovascular coupling is impaired. We used DOT to reconstruct images of $\Delta [{\mathrm{HbO}}_{2}]$, Δ[HHb], and Δ[oxCCO] from data acquired with a broadband system. Four healthy volunteers were measured while performing a visual stimulation task (4-Hz inverting checkerboard). The broadband system was configured to allow multidistance and overlapping measurements of the participants’ visual cortex with 32 channels. A multispectral approach was employed to reconstruct changes in concentration of the three chromophores during the visual stimulation. A clear and focused activation was reconstructed in the left occipital cortex of all participants. The difference between the residuals of the three-chromophore model and of the two-chromophore model (recovering only $\Delta [{\mathrm{HbO}}_{2}]$ and Δ[HHb]) exhibits a spectrum similar to that of oxCCO. These results form a basis for further studies aimed to further optimize image reconstruction of Δ[oxCCO].

## 1 Introduction

Near-infrared spectroscopy (NIRS) uses light in the red and near-infrared range to monitor concentration changes of oxy- (${\mathrm{HbO}}_{2}$) and deoxy- (HHb) hemoglobin in the brain.^1^ Under the assumption of an intact neurovascular coupling, these can be considered as an indirect measure of brain activity. When multiple sources and detectors are used, arranged in overlapping and multidistance channels, depth-resolved images of these cerebral hemoglobin variations can be reconstructed.^2^^,^^3^ This technique is usually referred to as diffuse optical tomography (DOT). Eggebrecht et al.^2^ have recently shown that by using a high-density DOT system, DOT can be employed to map distributed brain networks and function. Their maps disclosed a strong correspondence with the same maps obtained with functional magnetic resonance imaging (fMRI). Their results are extremely promising since DOT techniques are characterized by noninvasiveness and portability. They can be easily employed to monitor vulnerable subjects, such as neonates,^4^^–^^6^ patients with implanted devices,^2^ or critically ill patients who need bedside monitoring^7^ and who could not be previously monitored with fMRI. Both functional NIRS (fNIRS) and fMRI rely on the assumption of intact neurovascular coupling, although this assumption is frequently violated in cases of brain pathology.^8^^–^^11^ Pathology (e.g., acute brain injury, cerebral ischemia, and neurodegenerative disease) can alter the regional hemodynamic response to localized changes in metabolism and in extreme cases can lead to absence or inversion of the response. Although it is well recognized that an inverted fNIRS hemodynamic response [or an fMRI negative blood-oxygen-level dependent (BOLD) response] can be seen in both healthy volunteers and after brain injury, the exact physiological mechanisms underlying these responses remain unclear. Additional information about colocalized cerebral metabolic state, such as that provided by oxCCO, can help elucidate these pathways. Therefore, there is an unmet clinical need for a direct marker of cerebral metabolism.

Cytochrome c oxidase (CCO), the terminal enzyme in the mitochondrial respiratory chain, is responsible for more than 95% of cellular oxygen metabolism.^1^ The redox state of CCO can be measured with broadband NIRS^12^ and as such represents an opportunity to directly measure cellular oxidative metabolism. The change in the oxidation state of CCO (Δ[oxCCO]) reflects the balance between electron acceptance originating from nicotinamide adenine dinucleotide and donation to oxygen to form water, driving the mitochondrial proton electrochemical gradient and >90% of adenosine triphosphate production.^13^ The relationship between cerebral oxygen and metabolic substrate delivery and CCO has been extensively investigated in animals,^14^ neonatal asphyxia,^15^ adult brain injury,^16^ and healthy adults,^17^ including during functional activation.^18^ Importantly, Δ[oxCCO] is concordant with measurements of metabolism, including invasively measured lactate/pyruvate ratio^16^^,^^19^ and magnetic resonance spectroscopy-derived lactate.^15^ However, because the concentration of CCO is an order of magnitude lower than that of hemoglobin, an optimized optical technique to measure Δ[oxCCO] using broadband NIRS^20^^,^^21^ is typically required.

Present commercial fNIRS systems do not have the capability to measure Δ[oxCCO], and in-house systems developed explicitly to measure Δ[oxCCO] provide only a limited number of channels because of hardware cost and complexity, and therefore cannot perform DOT.^13^ Reconstructing images of Δ[oxCCO] is a natural progression of the technique following the development of DOT for displaying $\Delta [{\mathrm{HbO}}_{2}]$ and Δ[HHb]. Recovering the spatial distribution of this additional chromophore may well facilitate the investigation of cerebral energy status alongside functional activation, particularly in the context of brain pathology.^22^ However, the small Δ[oxCCO] signal that occurs in the presence of larger changes in $\Delta [{\mathrm{HbO}}_{2}]$ and Δ[HHb] means that imaging it is a significant challenge.

The recovery of images of concentration changes via DOT constitutes a nonlinear, ill-posed inverse problem.^23^^–^^25^ This process involves three steps. First, a geometric representation of the target object is required. A subject-specific, multilayer, anatomically accurate head model is preferred, but when the magnetic resonance image of the subject is unavailable, a multilayer atlas head model can be employed.^26^ The atlas head model can be registered to the subject’s cranial landmarks, and wavelength- and tissue-specific optical properties can be assigned to each tissue type [e.g., scalp, skull, cerebrospinal fluid (CSF), gray matter (GM), and white matter (WM)], thus providing an anatomically meaningful solution space. Second, a model of how light propagates through these head tissues, given an array of sources and detectors, is required, so as to relate changes in optical properties to changes in the acquired signals. This forward model can be constructed either with finite-element method (FEM) approaches (e.g., Toast++^27^ and Nirfast^28^) or with Monte Carlo approaches (e.g., MMC^29^ and MCX^30^). Third, the solution of the forward model must be inverted so as to solve the inverse problem and reconstruct an image of concentration changes. This step is the most computationally expensive, since it is an underdetermined and ill-posed problem. Corlu et al.^31^ demonstrated a multispectral approach, which directly reconstructs changes in concentration (rather than absorption changes at each wavelength). This approach reduces the number of unknowns and better constrains the inverse problem.

Most DOT studies to date have recovered images of $\Delta [{\mathrm{HbO}}_{2}]$, Δ[HHb], and of changes in total hemoglobin concentration Δ[HbT] (where $\Delta [\mathrm{HbT}]=\Delta [{\mathrm{HbO}}_{2}]+\Delta [\mathrm{HHb}])$, since these are the most studied chromophores in clinical and neuroscience settings.^2^^,^^4^^,^^5^ Other chromophores, such as water and lipids, have been successfully reconstructed with DOT,^31^^,^^32^ mostly for breast imaging applications.

The aim of this work is to provide, for the first time, a proof of concept demonstration of the feasibility of reconstructing images of oxCCO concentration changes, along with $\Delta [{\mathrm{HbO}}_{2}]$ and Δ[HHb], in the adult brain. Data were acquired from four healthy volunteers with a broadband system during a visual stimulation paradigm, and images were reconstructed with a multispectral approach. Visual stimulation was employed since it is known to elicit a strong localized activation with a highly repeatable pattern.^33^^,^^34^ Limitations and assumptions of the present work will be discussed.

## 2 Material and Methods

### 2.1 Instrumentation

A detailed description of the broadband system used in this study can be found elsewhere.^18^ The system has two 50-W halogen light sources, each delivering light to the participant via optical fibers. Only one source can be illuminated at a time. To collect the diffusely reflected light and measure the intensity at each wavelength, the system has eight detector fibers divided into two bundles and two CCD cameras (Pixis 512, Princeton Instruments, Trenton, New Jersey), each connected to a spectrograph. The sampling frequency is 1 Hz.

**Fig. 1:**
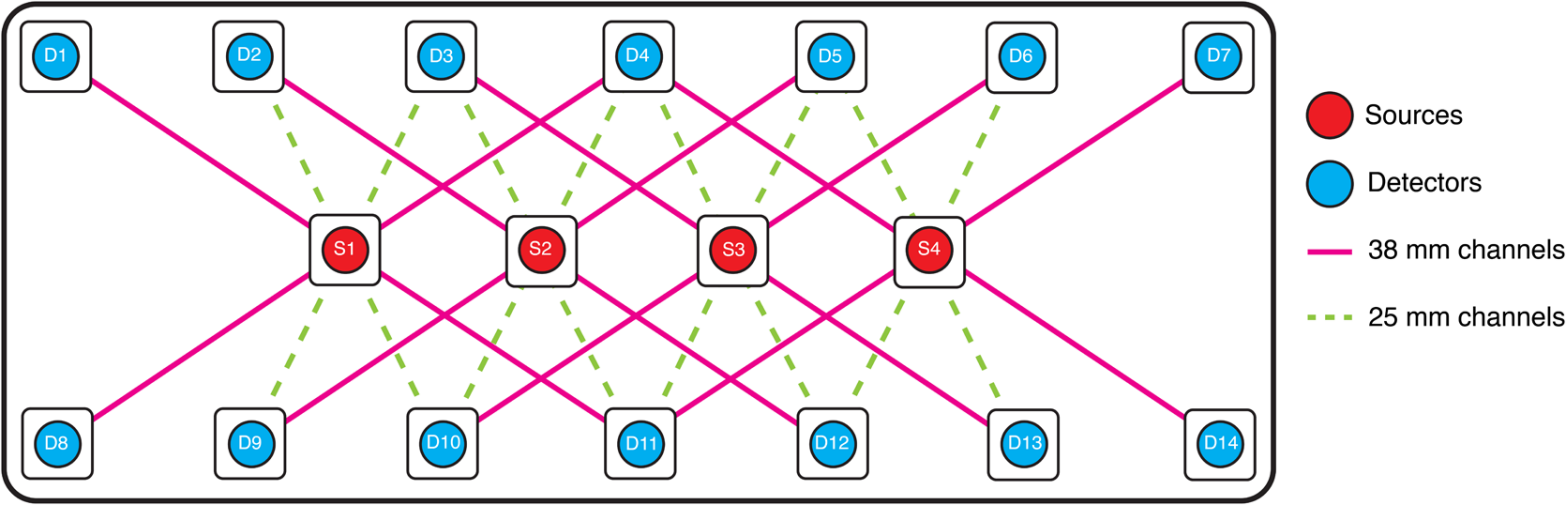
Array layout. Sources were positioned in the central row (red dots), while detectors were located in the bottom and top rows (blue dots). Eight channels per source could be recorded, for a total of 32 channels. Note that not all slots could be filled with a source or a detector during a single acquisition, since only one source and eight detectors were available at a time. Each acquisition was repeated four times, with the available source and detector fibers positioned in different slots for each acquisition so as to cover the entire imaging array over four acquisitions in each subject.

The optical interface between the system and the participant was based on a three-dimensional (3-D)-printed optode holder. The optode holder incorporated 4 source slots (in the middle row) and 14 detector slots arranged in 2 rows (see Fig. 1).

### 2.2 Participants and Paradigm

**Fig. 2:**
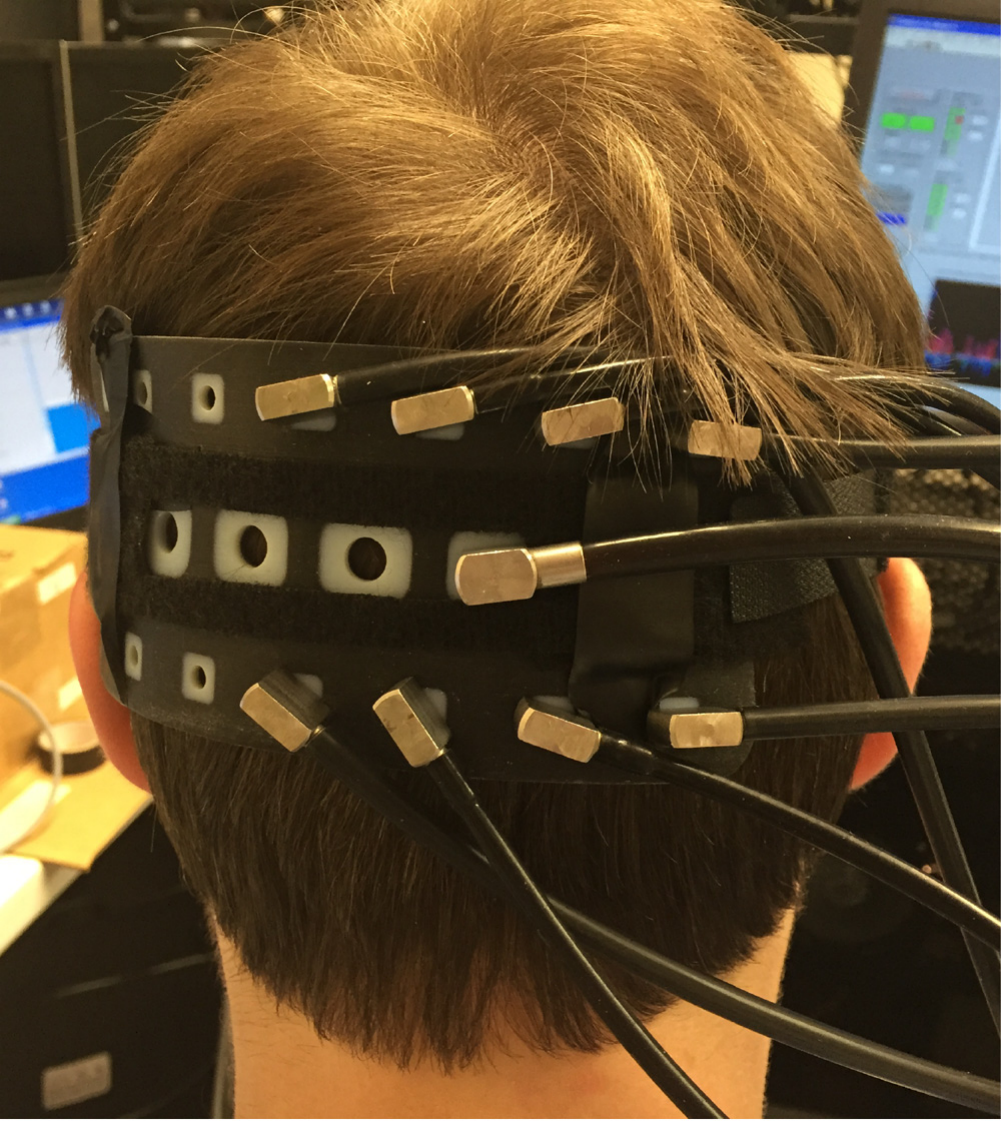
Optode holder placed on the left occipital cortex with one source (central row) and eight detectors.

Four healthy volunteers (two males and two females; age range 27 to 48 years old) participated in the visual stimulation study after providing written informed consent. Participants were seated in a comfortable chair in a dimly lit room and were asked to focus their attention at the center of the computer monitor, located at a distance of 60 cm in front of the subject. Visual functional activation was achieved with a 4-Hz inverting checkerboard covering the full visual field. The paradigm consisted of 20 s of stimulation followed by 20 s of blank screen repeated over 10 epochs. To monitor the visual cortex, the optode array was fixed horizontally to cover the participant’s left occipital cortex, with the fourth source location positioned over Oz (according to the 10-20 EEG positions) (Fig. 2). The eight nearest detector slots in the array (four above and four below each activated source) were populated with the eight available detector fibers and were used to collect intensity data continuously during the 10 epochs of visual stimulation. The remaining detector slots remained empty during that acquisition. This was repeated for each of the four source slots by translating the fibers over the fixed optode array on the head and repeating the stimulation. A 3-D Patriot^™^ Digitizer (Polhemus, Colchester, Vermont) was employed to acquire, for each participant, the coordinates of their cranial landmarks (nasion, inion, preauricular points, and Cz) in addition to the optode locations. The study was approved by University College London's Research Ethics Committee (1133/001).

### 2.3 Data Analysis

Attenuation changes ($\Delta A_{\lambda }$) were calculated from the intensity changes across each of the wavelengths from 740 to 900 nm collected during the challenge using the following equation:

$$\Delta A_{\lambda_{i}}=\log_{10}(\frac{I_{0}}{I_{\lambda_{i}}}),$$ (1)

where $I_{0}$ is an arbitrary reference intensity (set to $10^{5}$) and $I_{\lambda i}$ is the intensity collected by the detector at wavelength $\lambda_{i}$.

The 400-s trace of attenuation changes in each channel and for each wavelength was bandpass filtered (fifth-order Butterworth filter, cutoff frequencies: 0.01 to 0.25 Hz) and then block-averaged according to the timestamp of the stimulation to produce a 40-s trace of mean attenuation changes.

Channel-wise $\Delta [{\mathrm{HbO}}_{2}]$, Δ[HHb], and Δ[oxCCO] concentrations were computed by applying the Beer–Lambert law as implemented in the UCLn algorithm,^20^ as is commonly performed in spectroscopy. The wavelength dependence of the differential pathlength factor, as computed by Kolyva et al.,^18^ was taken into account when solving the Beer–Lambert law. These channel-wise data were recovered to show the results of the standard analysis employed in spectroscopy and compare them with the recovered images.

To reduce the computational burden of the reconstruction while covering the available near-infrared spectrum, attenuation changes measured at 17 discrete wavelengths (from 740 to 900 nm at intervals of 10 nm) were derived from the measured broadband data to perform the reconstruction.

### 2.4 Image Reconstruction Procedure

#### 2.4.1 Head model

The nonlinear MNI-ICBM152 atlas^35^ was the basis of our head model, which was built as described in Brigadoi and Cooper^36^ and Dempsey et al.^37^ A multilayer tissue mask was created by segmenting the MR image into five tissue types (scalp, skull, CSF, GM, and WM). A high-resolution tetrahedral mesh ($∼2\times 10^{5}$ nodes and $∼10^{6}$ elements) was created with the iso2mesh software.^38^ The same software was used to create the GM surface mesh ($∼3\times 10^{4}$ nodes and $∼6\times 10^{4}$ faces), which was used to display the reconstructed images.

The head model was registered to each participant’s head with an affine registration using the measured cranial landmarks as reference. The affine registration was also used to register the GM surface mesh.

#### 2.4.2 Inverse problem and image reconstruction

Images representing $\Delta [{\mathrm{HbO}}_{2}]$, Δ[HHb], and Δ[oxCCO] were reconstructed using a multispectral approach, which directly generates concentration changes from attenuation data.^31^ The multispectral approach is a standard approach,^31^^,^^37^ which has been shown to improve the accuracy of the reconstructed images as the number of wavelengths increases, as in our case. After linearizing the forward problem,^23^ we may relate changes in measured optical attenuation to changes in concentration through the multispectral Jacobian matrix

$$[\begin{array}{c} \Delta A_{\lambda_{1}}^{1}\\ \Delta A_{\lambda_{1}}^{2}\\ \cdots \\ \Delta A_{\lambda_{1}}^{n}\\ \Delta A_{\lambda_{2}}^{1}\\ \cdots \\ \Delta A_{\lambda_{17}}^{n} \end{array}]=[\begin{array}{ccc} J_{\lambda_{1}}\epsilon_{\lambda_{1}}^{{\mathrm{HbO}}_{2}} & J_{\lambda_{1}}\epsilon_{\lambda_{1}}^{\mathrm{HHb}} & J_{\lambda_{1}}\epsilon_{\lambda_{1}}^{\mathrm{oxCCO}}\\ J_{\lambda_{2}}\epsilon_{\lambda_{2}}^{{\mathrm{HbO}}_{2}} & J_{\lambda_{2}}\epsilon_{\lambda_{2}}^{\mathrm{HHb}} & J_{\lambda_{2}}\epsilon_{\lambda_{2}}^{\mathrm{oxCCO}}\\ & \cdots & \\ J_{\lambda_{17}}\epsilon_{\lambda_{17}}^{{\mathrm{HbO}}_{2}} & J_{\lambda_{17}}\epsilon_{\lambda_{17}}^{\mathrm{HHb}} & J_{\lambda_{17}}\epsilon_{\lambda_{17}}^{\mathrm{oxCCO}} \end{array}][\begin{array}{c} \Delta C_{{\mathrm{HbO}}_{2}}\\ \Delta C_{\mathrm{HHb}}\\ \Delta C_{\mathrm{oxCCO}} \end{array}],$$ (2)

where $\epsilon_{\lambda }$ is the specific absorption coefficient of ${\mathrm{HbO}}_{2}$, HHb, and oxCCO at wavelength $\lambda_{i}$, $J_{\lambda }$ is the wavelength-specific Jacobian matrix (number of channels × number of nodes), ΔC is the concentration change of ${\mathrm{HbO}}_{2}$, HHb, and oxCCO at each node, and ΔA is the attenuation change between active and rest state at each wavelength $\lambda_{i}$ and at each channel (from 1 to n).

Each wavelength-specific Jacobian was computed with the Toast++ software,^27^ which solves the diffusion approximation via the FEM. The Jacobian was computed on the volumetric head mesh. Optical properties (absorption coefficient and scattering coefficient) were assigned to each tissue type and for each wavelength by fitting all published values for these tissue types.^39^^–^^41^

The forward model solution was projected by Toast++ onto a 50×60×50 voxel regular grid for reconstruction, and an intermediate finer grid of 100×120×100 voxels was used to optimize the mapping between the mesh space and the voxel space. Diffuse boundary sources and detectors were simulated as a Gaussian profile with a 2-mm standard deviation, and Neumann boundary conditions were applied.

To solve the changes in concentration in a least squares-sense, we minimized the form

$$E(\Delta C)={\left\|\Delta A-J\Delta C\right\|}_{\Gamma_{e}^{-1}}^{2}+\lambda R(\Delta C),$$ (3)

where R(ΔC) is a suitable regularization functional whose influence is weighted by the hyperparameter λ, and $\Gamma_{e}^{-1}\;$is the inverse noise covariance matrix (set here as identity matrix).

The regularization functional R(ΔC) imposes prior knowledge of the solution to overcome the ill-posedness of the inverse problem. In this work, we chose to employ first-order Tikhonov regularization such that

$$R(\Delta C)={\left\|\nabla (\Delta C)\right\|}_{\Gamma_{x}^{-1}}^{2},$$ (4)

with the inverse parameter covariance matrix

$$\Gamma_{x}^{-1}=[\begin{array}{ccc} {\begin{array}{c} \left(C_{{\mathrm{HbO}}_{2}}^{b}\right) \end{array}}^{-2} & 0 & 0\\ 0 & {\begin{array}{c} \left(C_{\mathrm{HHb}}^{b}\right) \end{array}}^{-2} & 0\\ 0 & 0 & {\begin{array}{c} \left(C_{\mathrm{oxCCO}}^{b}\right) \end{array}}^{-2} \end{array}],$$ (5)

in which $C^{b}$ is the square matrix (number of voxels × number of voxels) with the background concentration values of the three chromophores: 56 for ${\mathrm{HbO}}_{2}$, 24 for HHb, and 12.8 for oxCCO^42^ on their diagonal. $C^{b}$ for oxCCO was set to 12.8 (instead of the suggested 6.4), given the higher concentration of oxCCO in the visual cortex.^43^ Use of the parameter covariance matrix spheres the solution space, thus ensuring that regularization is applied equally to each of the parameters, irrespective of their background concentrations. This is analogous to the normalization usually employed when absorption and scattering coefficients have to be simultaneously recovered.^23^^,^^44^ We employed first-order Tikhonov regularization to avoid suppressing the solution, thus ensuring an unbiased estimation of the three chromophore concentrations.

The LSQR method^45^ was employed to solve the matrix equations resulting from the minimization. For each reconstruction, the LSQR algorithm was configured to terminate after a maximum of 50 iterations, and with a tolerance of $\;10^{-5}$. The regularization hyperparameter was set to $\lambda =10^{-4}$ by inspection, after examining all images recovered varying λ from $10^{-2}$ to $10^{-5}$, and further confirmed by an L-curve analysis, as the highest point of curvature of the L-curve in each participant. The LSQR method is an iterative method, which has been proven to be analytically equivalent to the more well-known conjugate gradient, but with better numerical properties.^45^ Furthermore, it is a suitable method to solve the inverse problem when the Jacobian matrix is large and sparse, as in our case. By employing the LSQR method and a preconditioning technique,^23^ we could also reduce the amount of memory required by the system to solve the inverse problem, when compared to the standard Moore–Penrose inverse technique.^24^

The reconstructed images were defined on the same regular grid as the Jacobian. A remapping procedure was therefore performed to map back the reconstructed images from the voxel space to the volumetric head mesh. The volumetric head mesh-based image was then projected to the GM surface mesh by assigning a value to each node on the GM boundary surface that was equal to the mean value of all the volumetric mesh node values within a 3-mm radius.

For comparison purposes, the same procedure described above was employed to reconstruct images of $\Delta [{\mathrm{HbO}}_{2}]$ and Δ[HHb] only. In this case, a two-chromophore model was employed in the reconstruction, instead of the three-chromophore model (with ${\mathrm{HbO}}_{2}$, HHb, and oxCCO) used previously.

### 2.5 Residual Computation

Residuals were computed to investigate the spectral changes due to the oxCCO chromophore. Channel-wise attenuation changes were deduced from the concentration changes in the voxel space (i.e., before their mapping to the volumetric mesh and then to the GM surface mesh, which was performed for visualization purposes only). Attenuation changes were determined for both the concentration changes computed using the three-chromophore model and the changes computed using the two-chromophore model by multiplying the concentration changes voxel-wise data with the multispectral Jacobian.

The difference between the three- and the two-chromophore fits was then investigated. Ideally, if the model explains the data, the residuals should be multivariate Gaussian noise with covariance $\Gamma_{e}$, i.e., the model accounts for all nonrandom variability in the data. Given that image reconstruction is an ill-posed inverse problem that requires regularization, it is likely that even if the model explains the data, the residuals would not equate to the covariance of the assumed noise model. However, a model that better explains the data will have residuals that tend toward Gaussian noise. If the difference between the residuals of the two models has a nonrandom distribution across wavelengths, with the residuals of the three-chromophore model smaller than those of the two-chromophore models, this would suggest that the two-chromophore model is not accounting for a chromophore that is evident in the data. This analysis of residuals has been previously used in spectroscopy studies recovering $\Delta [{\mathrm{HbO}}_{2}]$, Δ[HHb], and Δ[oxCCO].^15^^,^^18^

Residuals were computed for each channel at the time of the peak of the expected functional activation (i.e., 20 s).

**Fig. 3:**
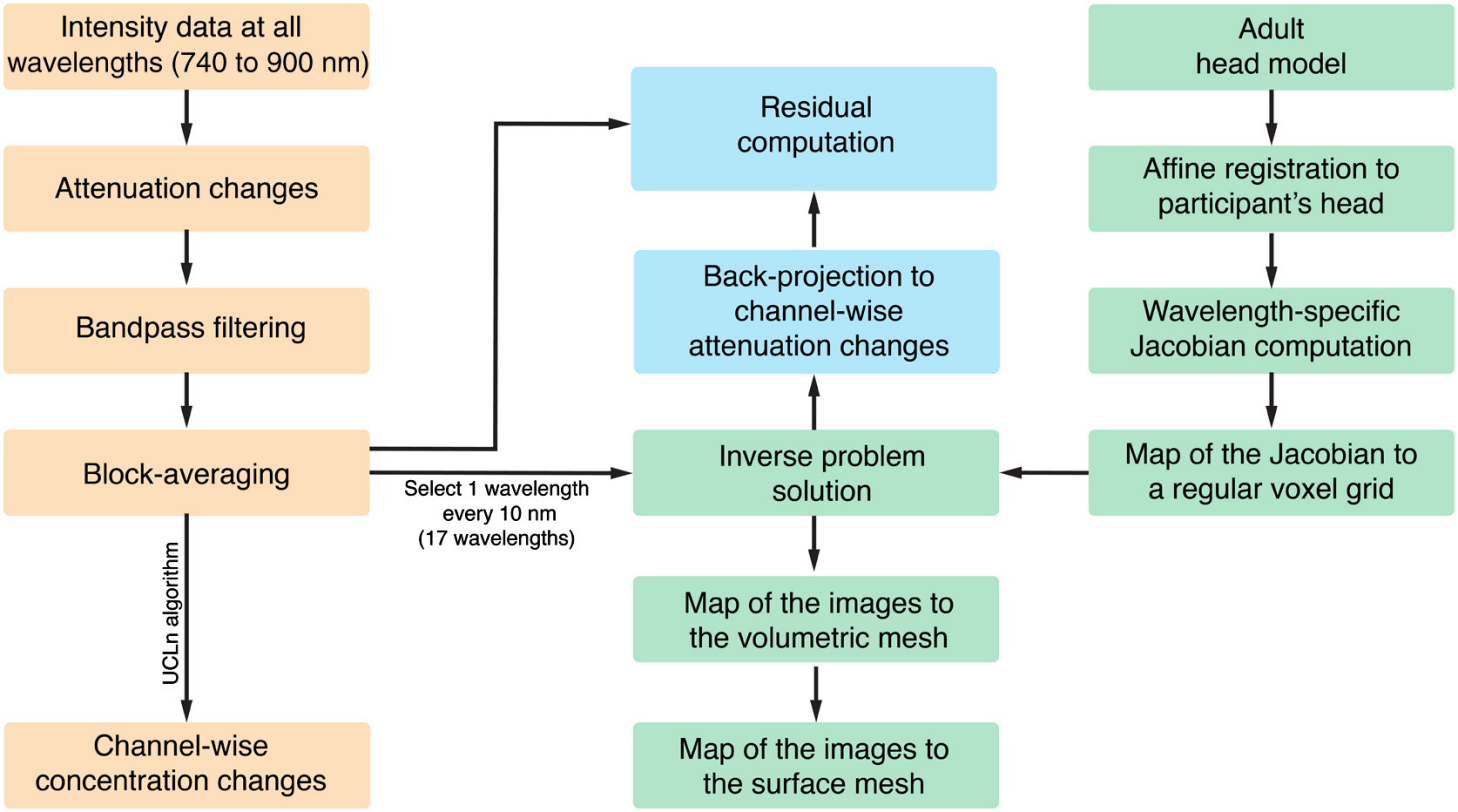
Flow diagram summarizing the main steps of the data analysis and image reconstruction procedures.

All analyses and reconstruction procedures were performed in MATLAB^®^ (Mathworks, Natick, Massachusetts). Figure 3 shows the data analysis and image reconstruction procedures.

## 3 Results

**Fig. 4:**
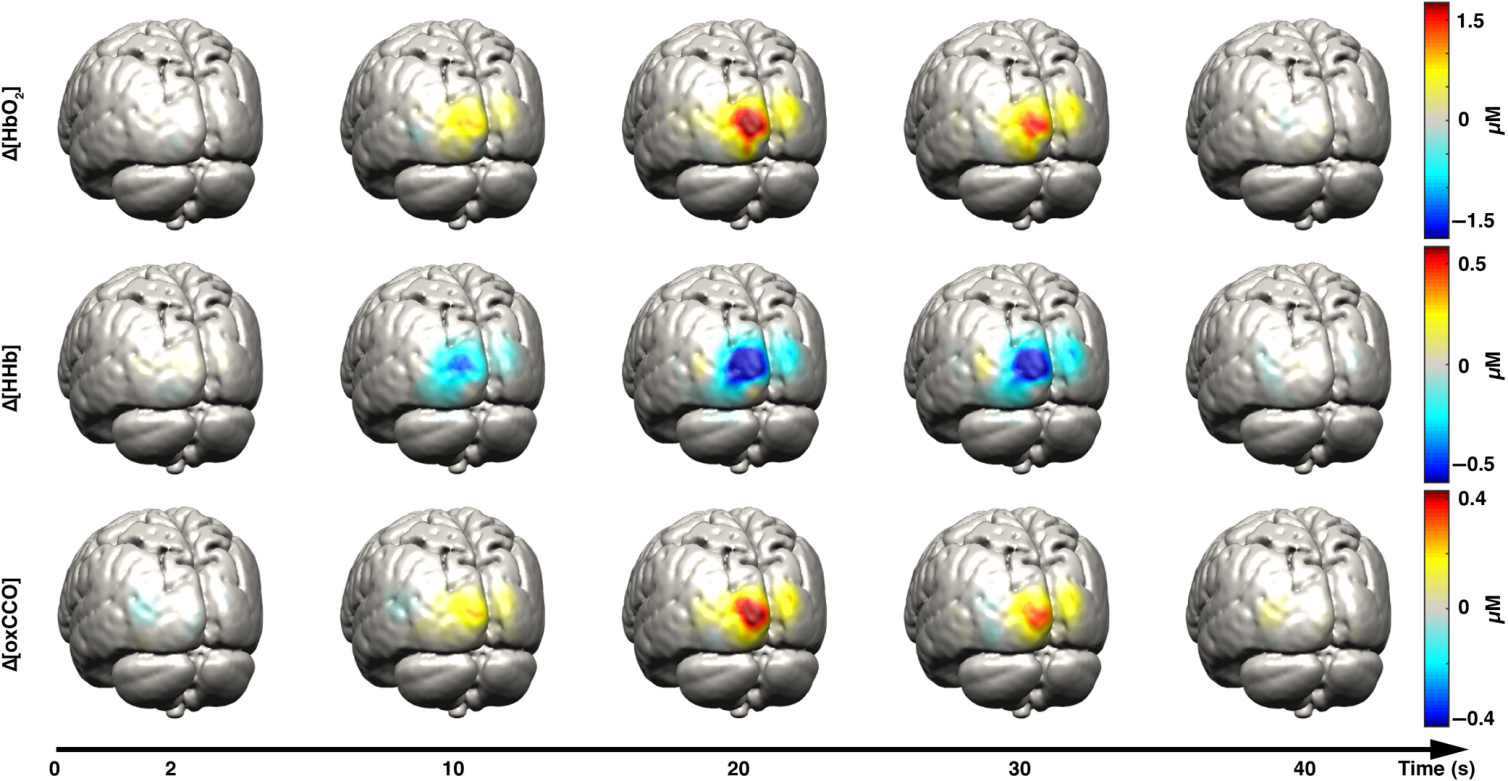
Examples of reconstructed $\Delta [{\mathrm{HbO}}_{2}]$ (first row), Δ[HHb] (second row), and Δ[oxCCO] (third row) images on the GM surface mesh at five different time points for participant 1. The selected time points are 2, 10, 20, 30, and 40 s after stimulus presentation. Images were mapped to the MNI152 GM mesh for visualization purposes.

**Fig. 5:**
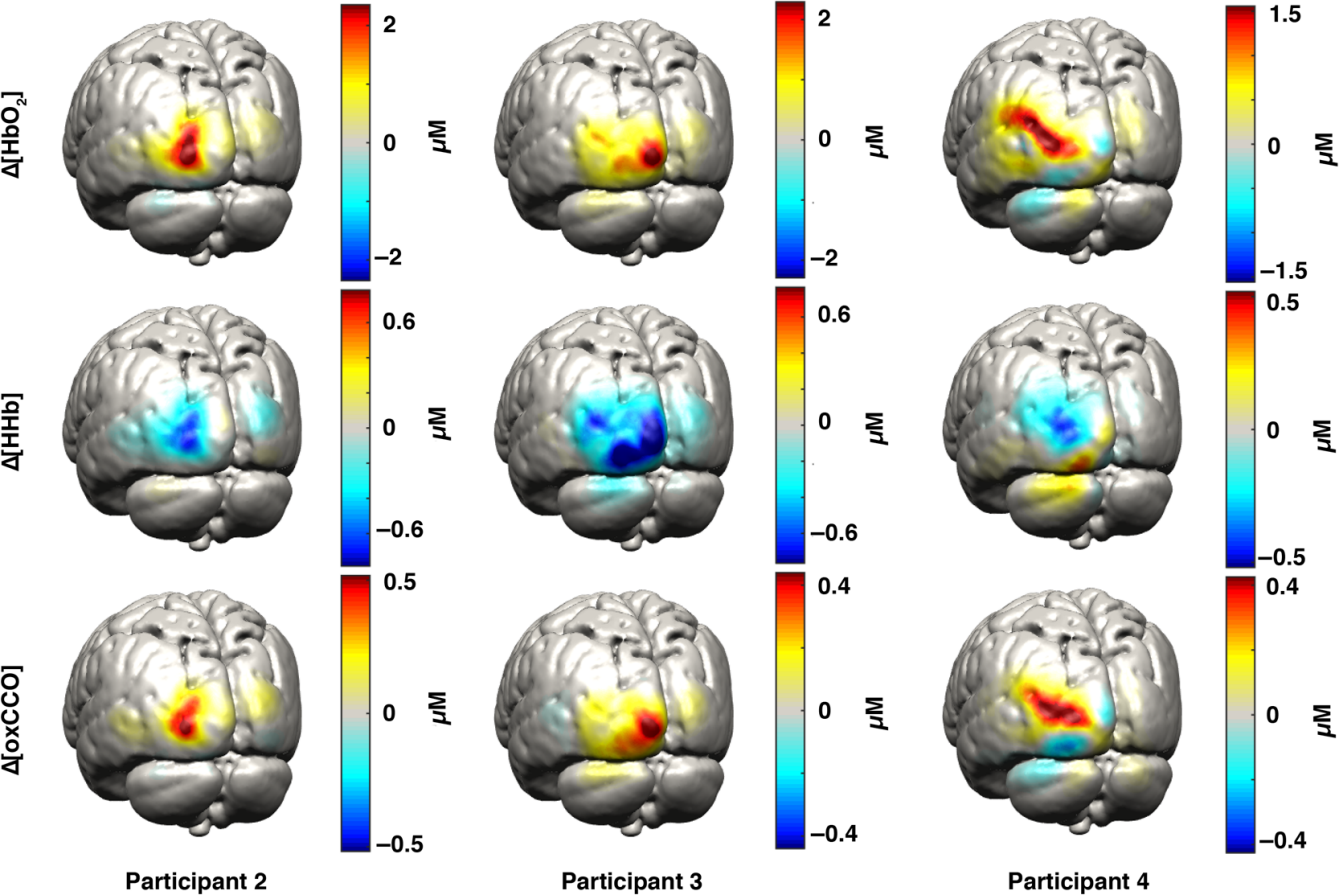
Examples of reconstructed $\Delta [{\mathrm{HbO}}_{2}]$ (first row), Δ[HHb] (second row), and Δ[oxCCO] (third row) images on the GM surface mesh for participant 2, 3, and 4. The reconstruction for 20-s poststimulus onset is displayed.

In Fig. 4, examples of reconstructed $\Delta [{\mathrm{HbO}}_{2}]$, Δ[HHb], and Δ[oxCCO] images on the GM surface mesh at five different time points are displayed for one representative participant (participant 1). The selected time points are 2, 10, 20, 30, and 40 s after stimulus presentation. Figure 5 shows the images obtained at 20-s poststimulus onset for the other three participants.

**Fig. 6:**
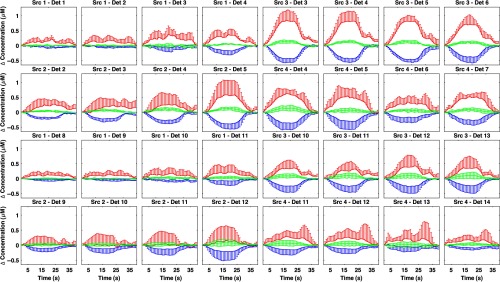
Grand-average hemodynamic responses in each channel to the functional activation task. Error bars represent the standard deviation across participants. Only the upper error bar for ${\mathrm{HbO}}_{2}$ and the lower error bar for HHb are reported for visualization purposes. $\Delta [{\mathrm{HbO}}_{2}]$ is reported in red, Δ[HHb] in blue, and Δ[oxCCO] in green. These responses were obtained using the data from the whole broadband spectrum (740 to 900 nm) and the UCLn algorithm.

Figure 6 shows the grand-average channel-wise recovered $\Delta [{\mathrm{HbO}}_{2}]$, Δ[HHb], and Δ[oxCCO] changes obtained using the spectroscopy approach over the whole 740- to 900-nm spectrum. Only some of the channels exhibit a strong activation (defined as an increase in ${\mathrm{HbO}}_{2}$ with a concomitant decrease in HHb), which is in line with the focal and localized change in chromophores reconstructed in Figs. 4 and 5.

**Fig. 7:**
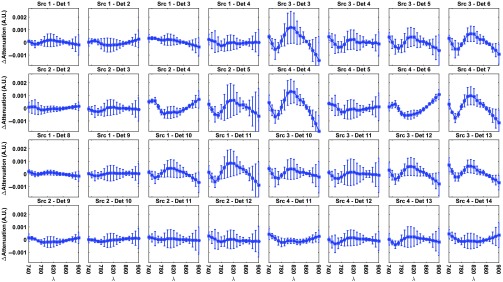
Grand-average residual difference between the two- and three-chromophore model in each channel and for each wavelength at time point 20. Error bars represent the standard deviation across participants.

Figure 7 shows the grand-average difference between the residuals of the two- and three-chromophore models in all channels. Residuals were computed as the differences between the measured data and the back-projected attenuation changes after image reconstruction. The spectra in most of the active channels do not have a random distribution. Instead, they show a shape approximating the spectrum of the oxCCO chromophore, with a broad peak at around 810 nm. In most of the channels that do not show activation, the difference between the residuals of the two models is almost nil. There is some variability in the residuals among participants (as there is in the hemodynamic responses, see Fig. 6) likely due to: (1) each participant can have a different timing of brain activation (all residuals are computed at time point 20 s) and (2) the retinotopic mapping of the visual cortex is different in each person, increasing the chance that each channel is probing slightly different brain visual areas in each participant.^46^

## 4 Discussion

In this study, we explored the feasibility of reconstructing images of Δ[oxCCO], in addition to $\Delta [{\mathrm{HbO}}_{2}]$ and Δ[HHb], using data acquired with a broadband NIRS system. This NIRS system allowed us to measure broadband data from overlapping and multidistance channels. With this configuration, we were able to perform DOT on broadband data and reconstruct images of Δ[oxCCO] for the first time. Data were acquired in four healthy volunteers during a visual stimulation task, which is known to elicit a strong activation, with a highly reproducible response.^33^^,^^34^

We evaluated the reconstructed images both qualitatively and quantitatively. All four participants showed a clear and focused activation for the three chromophores in the left occipital cortex during the period of maximum response to the visual stimulation. These results are in line with the channel-wise spectroscopy data, which show a clear response to the task in a subset of channels. Furthermore, by employing a two-chromophore model, we reconstructed from the same data images of $\Delta [{\mathrm{HbO}}_{2}]$ and Δ[HHb] only. We then computed the residuals for both the two- and the three-chromophore models as the difference between the original attenuation changes and the back-projected attenuation changes. The difference between these residuals provides insight on whether a third chromophore is apparent in the data. The residual difference spectra exhibited a shape similar to that of the oxCCO spectrum, a common finding in NIRS studies, thus suggesting that the functionally induced changes in oxCCO concentration are influencing our data and can be imaged via DOT.

It should be noted that while Δ[oxCCO], $\Delta [{\mathrm{HbO}}_{2}]$, and Δ[HHb] are normally recovered from NIRS data assuming a linear relationship between the changes in concentration and attenuation, in DOT, a nonlinear, ill-posed, underdetermined inverse problem must be solved. Therefore, the residuals in DOT should be interpreted with caution. Solving the inverse problem involves a balance between our trust in the data and our confidence in the prior knowledge. Residuals can be influenced by the fact that the problem is ill-posed and underdetermined, and by uncertainties in the prior knowledge. Indeed, the back-projected attenuation changes are unlikely to be identical to the measurements. Regularization is required to improve the conditioning of the inverse problem and to cope with noise amplification that can occur in the inversion process due to small singular values in the Jacobian matrix. First-order Tikhonov regularization was utilized here to avoid suppressing the mean value of the images and to prevent the build-up of excessive high-frequency components, which cannot be resolved using diffuse optical methods. By sphering the solution space prior to reconstruction, we ensured that the effect of the regularization was applied equally to each concentration value.

The optimal wavelength range for NIRS studies of functional changes in tissue is still under debate, 740 to 900 nm has been investigated,^34^ with preference for 780 to 900 nm to recover Δ[oxCCO], $\Delta [{\mathrm{HbO}}_{2}]$, and Δ[HHb]^15^^,^^18^ because of evidence of distortion over the peak of the HHb extinction spectrum between 740 and 780 nm. Arifler et al.^21^ reported the best combinations of wavelengths to recover Δ[oxCCO], $\Delta [{\mathrm{HbO}}_{2}]$, and Δ[HHb] in spectroscopy when the full broadband spectrum cannot be acquired. With a combination of three wavelengths, the mean recovery error versus the gold standard (i.e., using the full spectrum) was 10%, which was reduced to 2% when using a combination of eight wavelengths. In all combinations, the lowest selected wavelength was around 784 to 785 nm. However, previous DOT studies aimed at recovering only $\Delta [{\mathrm{HbO}}_{2}]$, and Δ[HHb] found that the best combination of wavelengths contained at least one wavelength below 780 nm, where measurements are more sensitive to HHb.^37^^,^^42^ Further studies should be performed to evaluate which wavelength combination is optimum to recover all three chromophores with DOT and to further investigate this issue. In this study, we selected 17 wavelengths from the broadband spectrum, separated at intervals of 10 nm. This choice was a compromise between the computational burden and the accuracy of the reconstructed image^32^^,^^47^ when using the multispectral approach proposed by Corlu et al.^31^ The computation time for the inversion process for one frame was ∼101  s with an Intel^®^ Core^™^ processor i7-4790 at 3.60 GHz.

Previous studies have shown the feasibility of recovering DOT images of $\Delta [{\mathrm{HbO}}_{2}]$ and Δ[HHb] in real time.^25^^,^^48^ Real-time imaging could provide valuable clinical information, which can be more naturally interpreted by clinicians. Considering the clinical importance of oxCCO, delivering real-time imaging of Δ[oxCCO], $\Delta [{\mathrm{HbO}}_{2}]$, and Δ[HHb] would be a great advance in the optical imaging field. Although from a theoretical perspective this should be readily achievable, there will likely be challenges to overcome when recovering images of all three chromophores in real time, mainly due to the higher number of wavelengths required and the associated increase in computational burden. Further studies should investigate the optimal number of wavelengths that can reliably yield Δ[oxCCO] images, and whether the computational burden of the reconstruction could be reduced to allow images of Δ[oxCCO], $\Delta [{\mathrm{HbO}}_{2}]$, and Δ[HHb] to be delivered in real time, perhaps using a dynamic Kalman filter estimator.^49^^,^^50^

This initial study will hopefully promote further research to optimize image reconstruction methods to deliver oxCCO changes and inform instrument development. Further work is required to explore the validity of the assumptions made in this study (e.g., the prior knowledge on the model covariance matrix, the selected wavelengths, the settings employed to solve the inverse problem, etc.), and the image reconstruction would benefit from further validation with simulations, and with phantom and *in vivo* studies. Ideally, the procedure should be verified using pathological conditions that induce changes in oxCCO but not in ${\mathrm{HbO}}_{2}$ or HHb concentration, which would reveal evidence of cross talk during the reconstruction of the three chromophores. Perhaps this could be achieved using an animal model of cardiopulmonary bypass with fluorocarbon perfusion.^51^^,^^52^

In this study, we have demonstrated for the first time the reconstruction of images of simultaneous changes in concentration of oxCCO, ${\mathrm{HbO}}_{2}$, and HHb in the healthy adult brain during a visual stimulation task. A clear and focal activation was reconstructed in the left occipital cortex for all three chromophores in all participants. Reconstructing images of Δ[oxCCO] may provide a unique opportunity to investigate cerebral energy status in addition to functional activation, across the cortex, which may have particular implications for studies of brain pathology.
